# Dissociative fugue in the elderly

**DOI:** 10.4103/0019-5545.58300

**Published:** 2009

**Authors:** Anita Rajah, R. Suresh Kumar, C. P. Somasundaram, A. Anand Kumar

**Affiliations:** Department of Clinical Psychology, Amrita Institute of Medical Sciences, Elamakkara, Kochi, Kerala, India; 1 Department of Neurology, Amrita Institute of Medical Sciences, Elamakkara, Kochi, Kerala, India

**Keywords:** Dissociation, fugue, memory

## Abstract

Dissociative fugue is a rarely reported diagnostic entity. It is one of the least understood and yet clinically one of the most fascinating disorders in Mental Health. Here, we describe a case of fugue in a 62-year-old housewife who was brought to our hospital with pockets of memory loss. This case illustrates the need for timely referrals, which could channelize valuable professional time and help avoid expensive and unnecessary investigations.

## INTRODUCTION

Dissociation is a protective activation of altered states of consciousness in reaction to overwhelming psychological trauma.[1]

DSM IV criteria for fugue require that the predominant disturbance is sudden, with unexpected travel away from home or one's workplace, coupled with the inability to recall one's past. The symptoms must also cause clinically significant distress or impairment in social, occupational, or other important areas of functioning.[2] Fugue differs from other mental disorders in that the flight behavior is organized and purposeful. The prevalence of dissociative fugue has been estimated at 0.2%, but it is much more common in connection with wars, accidents, and natural disasters.[3] Most fugues are brief and self-limited, the impairment being mild and short-lived. Treatment involves methods such as hypnosis or drug-facilitated interviews.

The differential diagnosis includes other dissociative disorders, seizures disorder, amnestic disorder, schizophrenia, mania, dementia (Alzheimer's type), malingering, and factitious disorder.[1]

## CASE REPORT

A 62-year-old lady was found wandering in the premises of a famous temple town in Kerala. When the local police questioned her, she said that she had left her home a few days ago and would now like to return. She provided them with the telephone number and address of her residence based on which the police were able to reunite her with her family.

On her return, the family discovered that she quickly got back to her old household routine, but was unable to remember people (neighbors, local shopkeeper) and events that had taken place in the preceding weeks. She was also confused about where she had gone and what she had done when she left home, but was able to furnish proof that she had traveled by train to a temple in a neighboring state, where she had spent her childhood. As they could not understand her patches of memory loss, the family brought her for a neurological evaluation.

An interview revealed that she was treated in the outpatient unit of the hospital for a fall six weeks ago. She had fallen from the pillion seat of a scooter and had been brought to the Casualty Section for first aid. There was no loss of consciousness, headache, blurring of vision, or vomiting. She was evaluated as an in-patient for possible post-traumatic sequelae. Medical examination revealed that she was hypertensive, but not on regular treatment. A neurological evaluation at that stage revealed no anomalies in her cognition or behavior. The CT scan and profile on blood investigations were within normal limits.

On this follow up, the Mini Mental State Examination score was 27. With no neurological problems elicited on examination and investigation, a working diagnosis of pseudo-dementia was made and a course of anti-depressants was prescribed.

On follow up, there was improvement in her mood, but the amnesia persisted. She was therefore referred for evaluation, to the clinical psychologist.

In this patient, there were no neurological or behavioral symptoms in the immediate post-traumatic period, except for mild features of a depressive disorder. The question here would be whether absence of “positive findings” could truly rule out organic causes for such impulsivity in behavior. A referral to the mental health practitioner is sometimes a resisted step in the treatment process, especially in a country such as India. It is only when the investigations rule out organicity, that “psychogenicity” of a symptom is considered.

### Evaluation in clinical psychology

In appearance, she was an obese lady who appeared anxious and tearful. She was initially unwilling to answer questions and repeatedly said that she could not remember what had happened. Speech was slow, but relevant and coherent. Mood was depressed; there was guilt and ideas of hopelessness. There were no hallucinations or delusions. Apart from deficits in semantic memory, there were no anomalies in primary mental functions.

On account of her apparent distress while answering questions, sessions of hypnosis were attempted. However, we encountered resistance and therefore, a supportive client-centered approach was adopted, with which she appeared to be more comfortable.

Formal psychometric assessment was deferred, in favor of serial Mental State Examinations.

Over the next three sessions, she was able to recall incidents of the preceding weeks. The most distressing episode according to her was the argument she had had with her husband. She felt that despite decades of managing the household and coping with several psychosocial stressors, the husband was overly critical of her. She described the intense grief she experienced that had prompted her to walk away from home. She visited several local temples before boarding a train to a famous temple town. After a day's stay there, she traveled by train to a temple in a neighboring state. It was the first time she was traveling alone by train, and she reported that she traveled ticketless on certain parts of the travel. She reported that she did not plan her movements, but seemed to instinctively move from one place to the other. It was only upon her return to her home state, after a few days that her memory “returned” and she went up to a local policeman and asked for help. She reported feeling very guilty over her “flight,” but insisted that there was no deliberate attempt to cause distress to her family. She did not remember people whom she was in contact with, during the travel, but could produce some of the tickets she had used. She had a purse with some money, but had pawned jewellery to purchase clothes and a few other necessities for herself. On her return, she was also very perplexed about the identity of some of the people around her and did not understand why she could not identify them.

There had been no incidents of similar behavior in the past; there were no reports of seizures, somnambulism, or other parasomnias. There were no differences in behavior and cognitive functions following the injury. The family described her as a quiet, responsible individual who had efficiently managed the household. Prior to her flight, she had participated actively in a family function; on her return, despite several cues, she could not recall the event or her family members who had reunited after a long while.

We considered the diagnosis of fugue because of the organized flight from home with significant disruption of her social and occupational routine, as also the absence of pre-morbid neurological or psychiatric problems and the self-remitting course of the altered behavior. The amnesia noted on her return was episodic and circumscribed (restricted to a few select people). The odd points in the case were the age and the absence of severe psychological trauma that could have precipitated the flight.

As we report the case, the client has returned to her pre-morbid level of functioning and comes regularly for follow up. She continues to be on a mild course of anti-depressants and supportive therapy. We plan to monitor her cognitive and psychiatric status over regular follow-up sessions and taper the sessions gradually if she maintains this improvement.

## DISCUSSION

It is well known that traumatic experiences affect the processing of memory, especially at the encoding stage. They create discontinuities with prior experience, involve arousal of intense affect (fear, sadness, anger) and may create conflicting patterns of association.[4] These conflicting networks of information can lead to over-selectivity in attention and therefore, prevent a more balanced view of the world.

The psychoanalytic school talks of defense mechanisms that individuals use, to cope with traumatic memories. Dissociation is sometimes referred to as splitting, as these thoughts, emotions, sensations, and/or memories are “split off” from the integrated ego.[3]

Dissociative phenomena may have strong biological roots, with genetic influences, accounting for about 50% of the variance in twin studies.[5] The presence of smaller hippocampal and amygdala volumes in patients with dissociative identity disorder has been reported.[6] Neurobiological theories postulate that NMDA blockade decreases the inhibitory tone, leading to increased glutamate release and subsequent dissociative symptoms, which also accounts for the structural brain changes in the hippocampus found in MRI studies.[3]

The ethical dilemma in such instances would be, how much to investigate, and more pertinently, when to make a referral for adjunctive services. In a developing country with a growing geriatric population, who have limited financial access to health care, these are relevant issues for the clinician to think about.

Dissociative disorders continue to remain underdiagnosed, undertreated, and insufficiently respected.[4] The rarity may be one of underdiagnosis and with proper screening and diagnostic instrumentation, a much higher prevalence may be encountered.[7]

Understanding a dissociative experience can thus add to the richness of the clinical material, and would highlight the need for multi-disciplinary approaches to treatment.

## References

[1] Sharon I, Sharon R Dissociative disorders.

[2] American Psychiatric Association (1994). Diagnostic and statistical manual of mental disorders.

[3] Sadock BJ, Sadock VA (2005). Comprehensive textbook of psychiatry.

[4] Spiegel D (2006). Editorial: Recognising traumatic dissociation. Am J Psychiatry.

[5] Jang KL, Paris J, Zweig-Frank H, Livesley WJ (1998). Twin study of dissociative experience. J Nerv Ment Dis.

[6] Vermetten E, Schmahl C, Lindner S, Loewenstein RJ, Bremner JD (2006). Hippocampal and amygdalar volumes in dissociative identity disorder. Am J Psychiatry.

[7] Foote B, Smolin Y, Kaplan M, Legatt ME, Lipschitz D (2006). Prevalence of dissociative disorders in psychiatric outpatients. Am J Psychiatry.

